# PUB2/420: Supporting a "Virtual Medical Worlds Community" with the "Virtual Magazine Publisher" Software System

**DOI:** 10.2196/jmir.1.suppl1.e105

**Published:** 1999-09-19

**Authors:** A Emmen

**Affiliations:** ^1^Genias Benelux, AlmereThe Netherlands

**Keywords:** Publishing, Telemedicine, Software

## Abstract

**Introduction:**

Creating an Internet based community requires a supporting software and hardware infrastructure that enables community members and community organisers to easily enter and update information, apart from other aspects such as discussion organisation and virtual meeting places. Here we describe a software system, "Virtual Magazine Publisher" (VMP) for managing magazines, newsletters, journals, member profile pages and project description pages. Its application for "Virtual Medical Worlds Community", a group of organisations involved in telemedicine and underlying advanced technologies is described.

**Methods:**

The VMP software (http://www.hoise.com/vmp) was designed, with the following criteria in mind:

- It must be suited for almost any magazine and newsletter
- Authors and editors should be able to access it from anywhere-Authors should be guided with entry of their contribution
- Editors should be given enough tools to manage their publication in much the same way they are used to
- The content should be separated from the lay-out-Information should be entered once: no need for hand conversions.

When analysing magazines we found that most have a simple structure, apart from glossy magazines and a number of scientific journals, being subdivided in sections, and each section is subdivided in articles or items. The lay-out of the articles in the section follow the same rules. Each issue has the same look and feel. Information from an article is used in the contents of the magazine. The structure is captured in an SGML definition (DTD) with two types of elements:

- for the general structure: title, subtitle, several lead parts, main text, and reference section
- for meta information: author, editors, issues, attached files

The issues can be constructed automatically from the information contained in each article using templates. Multimedia files that belong to an articles can identified by an "attach" element. The publishing software can help the editor with an overview of everything that belongs to an article or an issue.

**Results:**

The system is used for a number of magazines and communities. The most elaborate is the "Virtual Medical Worlds Community", a group of organisations involved in telemedicine and underlying advanced technologies (http://www.hoise.com/vmw/vmwc). The main parts of this community are:

- A news magazine called Virtual Medical Worlds Magazine: an extended table of contents is send by e-mail. The full magazine is available on the Web.
- ITIS letters: a journal for publishing fast refereed results from telemedicine research.
- Member profiles with activities, projects, products etc.
- Calendar.

**Discussion:**

The current system is implemented using an article description based on an HTML-extension. The system is written mainly in PERL, Javascript, and a Java application. The fast take-up of XML allows to revert this situation: a new design will use XML to implement the article structure.

